# Pathogen-Specific Risk for Iterative Surgical Debridement in Orthopedic Infections: A Prospective Multicohort Analysis

**DOI:** 10.3390/jcm14248750

**Published:** 2025-12-10

**Authors:** Flamur Zendeli, Anna Jędrusik, Raymond O. Schaefer, David Albrecht, Michael Betz, Felix W. A. Waibel, Tanja Gröber, Nathalie Kühne, Sören Könneker, İlker Uçkay

**Affiliations:** 1 Department of Orthopedic Surgery, Balgrist University Hospital, 8008 Zurich, Switzerland; flamur.zendeli@balgrist.ch (F.Z.); raymond.schaefer@balgrist.ch (R.O.S.); david.albrecht@ksb.ch (D.A.); michael.betz@h-och.ch (M.B.); felix.waibel@balgrist.ch (F.W.A.W.); 2 Department of Infectious Diseases and Hospital Epidemiology, University Hospital Zürich, 8091 Zurich, Switzerland; anna.jedrusik@usz.ch; 3 Unit of Clinical and Applied Research, Balgrist University Hospital, 8008 Zurich, Switzerland; tanja.groeber@balgrist.ch (T.G.); nathalie.kuehne@balgrist.ch (N.K.); 4 Department of Plastic Surgery and Hand Surgery, University Hospital Zurich, 8091 Zurich, Switzerland; soeren.koenneker@usz.ch; 5 Infectiology, Balgrist University Hospital, University of Zurich, 8008 Zurich, Switzerland

**Keywords:** orthopedic infection, surgical debridement, multiple procedures

## Abstract

**Background/Objectives**: Almost all orthopedic infections require a combination of surgical debridement with targeted antimicrobial treatment. The number of debridement procedures may vary considerably between infection episodes. The case mix is large, and so-called “second looks” are frequent. We investigate which bacteria are associated with second looks to achieve local infection control. **Methods**: We used a composite database stemming from three prospective randomized controlled trials (RCTs) from 2019 to 2025 with protocoled surgical debridement (initial debridement). In these RCTs, we allowed additional debridement only in the case of persistent or progressive local infection. **Results**: Overall, 201/1067 (18.8%) orthopedic infections required multiple debridement procedures. The median number of additional debridement procedures was two across all pathogen groups, with a range of 2–8 interventions. Gram-negative pathogens revealed the highest risk for “second looks” (28.2%), followed by implant-related infections (25.4%). Cutibacteria yielded the lowest risk (11.7%). In the multivariable logistic regression model, Gram-negative infections (OR 2.04, 95%CI 1.20–3.47) and infected implants (OR 2.18, 95%CI 1.56–3.03) were independently associated with multiple interventions, in contrast to *Staphylococcus aureus*, Enterococci, or Streptococci. **Conclusions**: Gram-negative pathogens were significantly associated with the need for second looks in orthopedic infections. The findings support preoperative counseling, antibiotic stewardship, and operative planning for staged management in infection cases with a high risk of clinical failure. Trials registrations: NCT04048304, NCT04081792, NCT05499481.

## 1. Introduction

Orthopedic infections (bone, joint, implants) represent a significant challenge, both in their incidence, cost, and management. A key aspect of this complexity is the frequent need for multiple surgical interventions—re-debridement (second looks)—for adequate source control, particularly in implant-associated infections [1,2,3,4,5,6]. Yet the number of debridement procedures required to achieve local infection control varies across patients, centers, and infections, with consequences regarding morbidity, length of hospital stay, costs, wound problems, re-education, and recovery [7]. Sometimes, second looks result in infection with new and more resistant pathogens [8,9]. Indicators for a second look must be thoroughly weighed against all possible negative consequences. And yet, these indicators very frequently remain subjective from the perspective of the responsible surgeon and their experience.

Among a myriad of reasons, pathogen biology might be a plausible driver for second looks. Differences in their virulence, toxins, and antimicrobial resistance, as well as polymicrobial presentations, may influence the ease—or difficulty—of achieving source control [4,5] after the first debridement. Orthopedic surgeons often advocate that, for example, *Staphylococcus aureus* is independently linked to multiple debridement procedures, although solid scientific data are lacking. Due to this subjectivity, retrospective case–control studies cannot assess strictly microbiological associations with second looks, because the surgical indication for a second look can reveal a mix of many objective and subjective parameters such as the surgeon’s opinion, objective local worsening, the patient’s wishes, rapid availability of surgery slots, and insurance levels (among many more). As such, it is not possible to assess a past indication in sufficient detail from medical files. The questions concerning second looks must be addressed in prospective trials that statistically control their large case mix per randomization.

We aim to address this important scientific gap by analyzing whether there is a relationship between the number of surgical interventions and the type of intraoperative microbiology in the first debridement. Clarifying this could inform operative planning (extent of debridement, need for staged procedures), counseling on expected trajectories, antibiotic stewardship, and resource allocation. It may also help to benchmark quality across centers by adjusting for the case mix among adult orthopedic patients.

## 2. Materials and Methods

In our hospital, we ran three RCTs (SALATIO [10], SASI [11], and TECH [12] infection studies), randomizing the duration of postsurgical antibiotic treatment. In all RCTs, the scheduled number of surgical interventions is one. Only in the case of uncontrolled infection—defined as persistent local signs (erythema, purulent drainage) for approximatively more than 4 days despite appropriate antibiotic therapy or clinical worsening at any time (new purulence, extension of inflammation, systemic deterioration)—did the surgeons, in consensus with the infectiology team, performed additional debridement. In the case of more than three interventions, the participating patients were excluded from the per-protocol analyses of each RCT. For this study, we included all orthopedic infections included in the RCTs from 1 June 2019 to 31 May 2025. In this analysis, we used the intention-to-treat (ITT) population, including all randomized patients regardless of the number of interventions received. While the original RCTs excluded patients with >3 interventions from per-protocol (PP) analyses of antibiotic duration, we retained all patients since our research question specifically concerns the frequency of surgical debridement.

We pooled infections across all three RCTs for the primary analysis. This approach is justified because, (1) from a microbiological perspective, pathogens behave similarly regardless of anatomical location; (2) stratification by trial would substantially reduce the statistical power for pathogen-specific analyses; and (3) multivariate adjustment for implant status addresses the main source of clinical heterogeneity between infection types.

We diagnosed an “orthopedic infection” as concordant growth of the same bacterial pathogen(s) in a minimum of two deep intraoperative tissue or bone cultures, together with (and/or) clinical signs of infection (purulence, drainage or sinus tract, erythema, warmth, pain). Histology or radiologic evidence (e.g., osteomyelitis, fluid collections, inflammatory changes) were facultative for diagnosis. “Implant” denoted any foreign material, excluding temporary wires or external fixator pins outside of the infected area. For practical study reasons, we classified the operated sacral osteomyelitis cases as “orthopedic infections”. Importantly, we recorded the number of surgical debridement interventions only if they were for infection and not due to non-infectious primary reasons such as seroma or hematoma. Likewise, we excluded previously planned second looks for infection [13], open fractures [14], elective correction [15], or elective implant removal [7]. We also excluded surgery for infection recurrences or for infections, i.e., unplanned revisions for infection relapse after the end of the scheduled antibiotic treatment. Hence, in terms of infection, our second looks concerned persistent local infection under surgical and antimicrobial therapy.

### Study Objectives and Statistical Analyses

As the primary and sole objective, we linked the intraoperative microbiological results of the initial debridement to the risk for second looks due to infection. We performed group comparisons using the Pearson chi^2^ test or the Kruskal–Wallis test, as appropriate. For stratified analyses, we grouped the pathogens into eight clinically relevant categories, following established conventions in the orthopedic infection literature, as follows: (1) *S. aureus*, (2) coagulase-negative staphylococci (CoNS), (3) Cutibacteria, (4) Streptococci, (5) Enterococci, (6) other Gram-positive bacteria, (7) Gram-negative rods, and (8) polymicrobial infections (more than one pathogen identified in intraoperative tissue or bone cultures). In the second step, a multivariate logistic regression model was adjusted for the large case mix. The pathogen categories, which were considered mandatory, were included in this final model, with CoNS used as the reference category. Polymicrobial infections were analyzed separately because of inherent interaction with other categories. We performed all analyses using RStudio Version 4.3.1 (R Foundation for Statistical Computing, Vienna, Austria) and considered a *p*-value < 0.05 (two-tailed) as significant.

## 3. Results

We included 1067 independent orthopedic infections in adult patients into the composite database. Of those, 374 involved retained implants, 381 were osteomyelitis cases, 305 involved the spine, and 381 were diabetic foot infections. *S. aureus* (n = 202, 18.9%), CoNS (n = 174, 16.3%), Cutibacteria (n = 103, 9.7%), and Gram-negative organisms (n = 103, 9.7%) were the most frequent bacteria. Polymicrobial infections accounted for 283 cases (26.5%) (Table 1). Among the 283 polymicrobial infections, 164 (58.0%) contained at least one Gram-negative pathogen. The rate of second looks in polymicrobial infections with Gram-negative involvement was 22.6% (37/164), compared to 19.3% (23/119) in polymicrobial infections without Gram-negative pathogens. Among the Gram-negative isolates, three were extended-spectrum beta-lactamase (ESBL) producers, and none demonstrated carbapenem resistance (CR). We could not perform stratified analysis by resistance phenotype due to the small sample size and anticipated underpowering. We did not assess the presence of small-colony variants among *S. aureus*, because we would consider them a risk for ultimate relapse rather than as a risk for early second looks [16]. The Supplementary Materials Table S1 is an expansion of the Table 1 with more detailed insights into the microbiology of our composite study population.

Overall, 201 patients (18.8%) required a second look within 1 to 2 weeks after the first debridement. The median number of debridement interventions was one across all microbiological strata, with a range of 2–8 interventions. In group comparisons, the number of interventions varied across pathogen groups (Kruskal–Wallis test; *p* = 0.009). Gram-negative infections revealed the highest risk for second looks (28.2%), followed by Enterococci (22.2%) and polymicrobial infections (21.2%). The lowest risk yielded Cutibacteria (11.7%) (Table 2).

**Figure 1:**
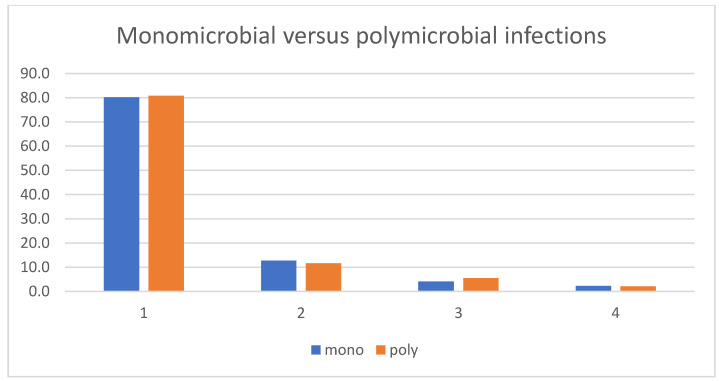
Number of debridement interventions in monomicrobial and polymicrobial infections.

After these first results were determined, we added direct binomial head-to-head comparisons between clinically important and frequent pathogen groups (Table 3). These second comparisons also showed a higher risk for second looks in Gram-negative infections compared to Gram-positive infections (28.2% vs. 17.1%, *p* = 0.001). Again, we observed no differences among the Gram-positive groups, such as between *S. aureus* and CoNS (19.3% vs. 16.7%, *p* = 0.51) or *S. aureus* against Streptococci (19.3% vs. 20.3%, *p* = 0.86). Overall, implant-related infections required a second look more often than native bone infections or those with removed implants (25.4% vs. 15.3%, *p* < 0.001) (Table 3). The number of debridement procedures between poly- and monomicrobial infections was similar (Table 3, Figure 1).

### Case Mix Adjustment

We added a logistic regression analysis with the outcome “second look” to adjust for the case mix. In its multivariate model, infected implants were independently associated with multiple debridement procedures (OR 2.18, 95%CI 1.56–3.03). However, regarding the pathogen groups, the largest odds ratios were observed for Gram-negative (OR 2.04, 95%CI 1.20–3.47) and polymicrobial infections (OR 1.73, 95%CI 1.13–2.65). The latter results reflected the Gram-negative involvement within the group of “polymicrobial infections”. Gram-positive bacteria such as *S. aureus*, CoNS, Cutibacteria, Streptococci, and Enterococci did not alter the risk for second looks (Table 4). Due to substantial heterogeneity within the Gram-negative pathogen group (Table 1) and anticipated underpowering due to further stratification among the Gram-negative bacteria, we were unable to dissociate the influence of (inherent) antibiotic resistance (among all Gram-negatives) in terms of early reinterventions. Among the Gram-positive bacteria, only less than 5% of *S. aureus* but the majority of CoNS were formally resistant to methicillin. Additionally, most CoNS were associated with implant infections.

## 4. Discussion

According to our single-center, composite prospective cohort with more than a thousand episodes of moderate-to-severe orthopedic infections, the indication for unplanned second looks was 18.8%. This overall risk significantly varied among all pathogen groups and revealed surprising findings. In striking opposition to our prior presumption, it was neither *S. aureus* [17,18] nor other Gram-positive bacteria such as Enterococci [19] or Streptococci [20] that predicted ultimate re-debridement. It was Gram-negative involvement and implant-related infections that doubled the odds of multiple debridement interventions (OR of 2.04 and 2.18, respectively). Nota bene, a persistently infected implant is not only a risk for infection recurrence [17,19,20] but also for a second surgical intervention to achieve local infection control. Polymicrobial infections tended to lead to re-interventions (albeit not significantly). However, we attributed this tendency to the Gram-negative bacteria in the polymicrobial infections. Unsurprisingly, the skin commensal Cutibacteria, which are the main cause of low-grade infections [21], yielded the lowest risk. We believe our findings have practical implications. Firstly, they may support counseling that Gram-negative involvement carries a higher likelihood of requiring more than one debridement. Secondly, they are in favor of proactive operative planning (e.g., awareness of staged procedures, broader irrigation/debridement) and an early reassessment when infected implants are retained. Thirdly, they can inform benchmarking, infection control, antibiotic stewardship, and quality improvement when comparing across services.

Our results are in line with existing trends and traditions. They re-confirm the indication to remove all infected implants to perform the best debridement possible (if feasible). Secondly, the “persistence” of Gram-negative bacteria against a single debridement follows a general trend in infectiology and healthcare epidemiology. Gram-negative infections have become more and more prevalent in virulent bacterial infections, including in orthopedic surgery such as for implant-associated spine infections [22] or community-acquired diabetic foot infections [23,24]. Gram-negative bacteria start to prevail over Gram-positive bacteria in many regions of the world [25,26], for which all reasons remain unsolved and represent a subject of ongoing debate. It is certain that Gram-negative infections parallel the epidemics of acquired or natural antimicrobial resistance and/or of systemic antibiotic use prior to hospitalization [27]. The prevalence of Gram-negative orthopedic infections and their antibiotic resistance is highly intermixed [24,26,28,29]. In our analysis, we could not stratify the natural resistance to link it with the risk of second looks because of the anticipated underpowering of the corresponding analyses. Moreover, a high proportion of resistant Gram-negative bacteria in our RCTs received broad-spectrum antibiotic coverage.

The scientific literature regarding our study question is very sparse, retrospective, and underpowered. The existing literature rather explores—mostly indirectly—the independent impact of specific pathogens on the final outcomes after therapy, with occasional notes of the number of surgical debridement procedures during treatment. The bulk of the available literature reports the DAIR approach for acute arthroplasty infections [4,5,6,30,31] (debridement, antibiotics, and implant retention when explanting intermediary spacers [32,33]) or fracture-related infection concerns [34,35,36], with few other surgeries [37,38]. Notably, in these retrospective papers, a “second look” is mentioned but not further investigated. To the best of our knowledge, no prospective study has addressed our question directly. Retrospectively, Chang and colleagues [6] reported that polymicrobial infections tend to be associated with failure in DAIR, although this difference did not reach statistical significance. Specifically, they defined DAIR failure as the need for repeated DAIR or a two-stage revision nota bene after the end of treatment for the index infection. Hence, their “second look” was again a DAIR approach. This paper is echoed by Chen [4] et al., finding a significant difference in DAIR failure between poly- and monomicrobial infections (55% vs. 21%). However, their cohort comprised only 106 arthroplasty infections in total, of which only nine were polymicrobial cases. In a much larger multicenter cohort study, Zhu [5] et al. identified *S. aureus* (OR 4.70) and Gram-negative pathogens (OR 2.56) as independent risk factors for DAIR failure again after the end of therapy. DiPaola et al. [39] developed a predictive model to stratify patients needing single vs. multiple debridement interventions in spinal infections. Positive MRSA (methicillin-resistant *S. aureus*) cultures (OR 2.71) represented the strongest predictor for multiple debridement procedures. In another study by Billières et al. [40], retrospectively analyzing spine infection, no variable was associated with remission. In particular, remission was unrelated to the number of second looks (hazard ratio 0.9; 95%CI 0.8–1.1), *S. aureus* (HR 0.9; 95%CI 0.8–1.1), intraosseous antibiotic use (HR 1.2; 0.6–2.4), or the total duration of antibiotic treatment (HR 1.0; 0.99–1.01) [40]. All these publications were retrospective, with difficulties in adjusting for the large case mix. All of them were rather underpowered, at least regarding the population size, for further stratified analyses.

The Wuarin study [8] bears the closest resemblance to ours. Their investigation, including a total of 2480 orthopedic infections, focused on repeated debridement interventions in relation to optimal perioperative prophylaxis during second looks. Overall, 1617 (65%) episodes were debrided once, compared to 862 cases with second looks (35%). Furthermore, this study underlines that “second looks” can become harmful. In terms of epidemiology, they can be an independent factor for new (and resistant) surgical site infections at the index site, despite ongoing antibiotic treatment for the index infection, especially when the second look (still) cannot close the wound. This secondary risk can reach 7% to 10% [8].

Our study has strengths and limitations. The major strength is its design. In our single center, we used the data of three prospective RCTs on antibiotic durations. The sample size of these RCTs surpasses 1000 infection episodes. Of importance is that, per protocol, the number of debridement procedures was fixed to one intervention, with second looks permitted in cases of obvious failure to control infection. A second strength is the experience of our orthopedic and plastic surgeons with orthopedic infections and with debridement. The main limitation is the heterogeneity of infection types (implant-related, native bone/joint, spine, diabetic foot infections), which may introduce clinical variability that is not fully captured by the current statistical models. Secondly, we only analyzed the risk for early second looks during therapy and not recurrence of infection after the treatment. In other words, our outcome was a clinically important early “persistence” of infection despite adequate antibiotic therapy and not ultimate failure. Both outcomes must not be confounded. For the first, almost no literature exists. For the latter, there is an abundance of scientific publications.

Third, the anatomical categories (osteomyelitis, spine, diabetic foot, implant-related) are not mutually exclusive, and overlap exists. We intentionally refrained from establishing a hierarchy to include all orthopedic infections and to avoid underpowering of the subgroup analyses.

Fourth, our classification of pathogens into eight categories, although based on established clinical and microbiological conventions, inevitably involves some degree of simplification. However, our eight categories follow established clinical and microbiological conventions used in the orthopedic infection literature, as follows: (1) *S. aureus* is universally analyzed separately due to its unique virulence and prevalence; (2) CoNS are distinct from *S. aureus* in pathogenicity; (3) Cutibacteria cause predominantly low-grade infections; (4–5) Streptococci and Enterococci have distinct clinical behaviors and virulence; (6) other Gram-positive bacteria are skin commensals prevalent in orthopedic infections; (7) Gram-negative rods share intrinsic resistance patterns and increasing prevalence; and (8) polymicrobial infections require separate analysis due to overlapping effects. Fifth, we could not analyze the independent effect of particular resistance phenotypes (ESBL, CRE, MRSA) due to small subgroup sizes, which represent an important area for future multicenter studies. Future multicenter studies should investigate whether specific Gram-negative resistance patterns (ESBL, CRE) differentially affect debridement requirements and whether standardized second-look criteria could reduce practice variation.

## 5. Conclusions

According to our recent prospective study including 1067 orthopedic infections in adult patients, the presence of Gram-negative pathogens (and not Gram-positive ones) and infected implants were associated with the need for “second looks”. This new information is useful for the counseling of patients, antibiotic stewardship issues, infection control, and planning surgical management. Ideally, every infected implant should be removed. We are confident in our findings. The main strength of our analysis is the use of a composite database of prospective interventional RCTs on antibiotic durations, for which we prohibited “second looks” unless there was a clear inevitable clinical indication. The main limitation is the single-center setting with a possible traditional bias among our surgeons. Prospective interventional trials in other settings are required to confirm or discard our results.

## Figures and Tables

**Table 1:** Pathogen distribution (n = 1067, 100%).

Gram-positive	532 (49.8%)		
*S. aureus*	202 (18.9%)	Coagulase-negative staphylococci	174 (16.3%)
Methicillin-sensitive	184	*S. epidermidis*	128
Methicillin-resistant	18	*S. lugdunensis*	18
		*S. caprae*	15
*Cutibacterium* spp.	103 (9.7%)	*S. hominis*	3
		*S. saccharolyticus*	2
*Streptococcus* spp.	34 (3.2%)	*S. capitis*	2
*S. pneumoniae*	3	*S. simulans*	1
*S. dysgalactiae*	7	*S. pseudointermedius*	1
*S. agalactiae*	10	*S. warneri*	1
*S. bovis*	2	*S. haemolyticus*	3
*S. gordonii*	2		
*S. anginosus*	2	*Enterococcus* spp.	9 (0.8%)
*S. sanguis*	1	*E. faecalis*	8
*S. mitis*	5	*E. faecium*	1
Other streptococci	2	Other Gram-positive	10 (0.9%)
Gram-negative	103 (9.7%)		
*P. aeruginosa*	25		
*Enterobacter* sp.	22		
*Escherichia coli*	19		
*Klebsiella* sp.	11		
*Proteus* sp.	10		
*Serratia marcescens*	7		
*Citrobacter* sp.	2		
Other	7		
Polymicrobial	283 (26.5%)		
Culture-negative	149 (14%)		

Values are expressed as absolute numbers with percentages in parentheses.

**Table 2:** Surgical intervention counts by pathogen group.

	Median Number of Surgical Interventions (Ranges) ^a^	Risk for Second Looks	95%CI ^b^
*S. aureus*	2 (2–6)	39/202 (19.3%)	14.5–25.3%
Coagulase-neg. staphylococci	2 (2–8)	29/174 (16.7%)	11.9–22.9%
*Cutibacterium* spp.	2 (2–4)	12/103 (11.7%)	6.8–19.3%
*Streptococcus* spp.	2 (2–4)	7/34 (20.6%)	10.3–36.8%
*Enterococcus* spp.	2 (2–2)	2/9 (22.2%)	6.3–54.7%
Other Gram-positive	2 (2–2)	2/10 (20%)	5.7–51.0%
Gram-negative	2 (2–8)	29/103 (28.2%)	20.4–37.5%
Polymicrobial	2 (2–5)	60/283 (21.2%)	16.8–26.3%
Culture-negative	2 (2–7)	21/149 (14.1%)	9.4–20.6%

^a^ Values expressed as the median with the range in parentheses. ^b^ Confidence intervals for proportions were calculated using the Wilson scoring method.

**Table 3:** Head-to-head comparisons between pathogen groups.

	Single Debridement	Multiple Procedures	*p*-Value ^c^
Gram-positive infections vs. Gram-negative infections	441/532 (82.9%)	91/532 (17.1%)	
			**0.001**
	74/103 (71.8%)	29/103 (28.2%)	
Monomicrobial vs. Polymicrobial infections	515/635 (81.1%)	120/635 (18.9%)	
			0.42
	223/283 (78.8%)	60/283 (21.2%)	
*S. aureus* vs. Coagulase-negative staphylococci	163 /202 (80.7%)	39/202 (19.3%)	
			0.51
	145/ 174 (83.3%)	29/174 (16.7%)	
*S. aureus* vs. *Streptococcus* spp.	163 /202 (80.7%)	39/202 (19.3%)	
			0.86
	27/34 (79.4%)	7/34 (20.6%)	
Implant-related infections vs. Native infections	279/374 (74.6%)	95/374 (25.4%)	
			**0.00001**
	587/693 (84.7%)	106/693 (15.3%)	

A *p*-value  < 0.05 was considered significant (in bold). ^c^ The Pearson chi^2^ test was used to analyze the differences.

**Table 4:** Multivariable logistic regression: predictors for a second look.

Parameter	Odds Ratio	95%CI	*p*-Value
Implant-related infections	2.18	1.56–3.03	**<0.001**
*Cutibacterium* spp.	0.57	0.29–1.14	0.113
Gram-negative bacteria	2.04	1.20–3.47	**0.009**
*S. aureus*	1.38	0.86–2.20	0.18
Coagulase-negative staphylococci	1.00	0.53–1.83	0.98
*Streptococcus* spp.	1.22	0.50–2.99	0.662
*Enterococcus* spp.	1.25	0.25–6.28	0.79
Polymicrobial	1.73	1.13–2.65	**0.012**
Other Gram-positive bacteria	1.23	0.25–6.08	0.799
Culture-negative infections	1.22	0.24–6.27	0.83

A *p*-value  < 0.05 was considered significant (in bold).

## Data Availability

We may provide anonymized key variables upon reasonable scientific request to the corresponding author.

## Supplementary Materials

The following supporting information can be downloaded at: https://www.mdpi.com/article/10.3390/jcm14248750/s1, Table S1: List of the cause pathogens with the crude incidences for a surgical second look.

## Abbreviations

The following abbreviations are used in this manuscript:

CoNS	Coagulase-negative Staphylococci
RCT	Randomized clinical trial
DFI	Diabetic foot infection
SSI	Surgical site infection
DAIR	Debridement, antibiotics, and implant retention
