# Supplementary material for: Pathogen-Specific Risk for Iterative Surgical Debridement in Orthopedic Infections: A Prospective Multicohort Analysis
Source: J Clin Med. 2025 Dec 10;14(24):8750. doi: 10.3390/jcm14248750 (PMC12733961; doi:10.3390/jcm14248750)
Supplement: Supplementary file 1 [file jcm-14-08750-s001.zip › jcm-4001643-supplementary.pdf]

Gram-positives	No.	>2 debridements	%	Gram-negatives	No.	> 2 debridements	%
**Coagulase-negative staphylococci**							
S. epidermidis	128	23	17.97	*Pseudomonas aeruginosa*	25	13	52.00
S. lugdunensis	18	1	5.56	*Enterobacter* spp	22	6	27.27
S. caprae	15	1	6.67	*Escherichia coli*	19	3	15.79
S. hominis	3	1	33.33	*Klebsiella* spp	11	4	36.36
S. saccharolyticus	2	0		*Proteus* spp	10	1	10.00
S. capitis	2	1	50	*Serratia* spp	7	1	14.29
S. simulans	1	0		*Citrobacter* spp	2	1	50.00
S. pseudointermedius	1	0		Other	7	0	
S. warneri	1	0					
S. haemolyticus	3	2	66.67				
Staphylococcus aureus							
S. aureus (methicillin-susceptible)	184	33	17.93				
S. aureus (methicillin-resistant)	18	6	33.33				
**Enterococci**							
E. faecalis	8	1	12.50				
E. faecium	1	1	100.00				
**Streptococci**							
S. pneumoniae	3	0					
S. dysgalactiae	7	2	28.57				
S. agalactiae	10	2	20.00				
S. bovis	2	0					
S. gordonii	2	1	50.00				
S. anginosus	2	1	50.00				
S. sanguis	1	0					
S. mitis	5	1	25.00				
Other streptococci	2	0					
Cutibacterium *sp.*	103	12	11.7				

Supplementary Table S1 - List of the cause pathogens with the crude incidences for a surgical second look
